# Psoriasis vulgaris flare during efalizumab therapy does not preclude future use: a case series

**DOI:** 10.1186/1471-5945-5-9

**Published:** 2005-08-18

**Authors:** Michelle A Lowes, James A Turton, James G Krueger, Ross StC Barnetson

**Affiliations:** 1 Laboratory for Investigative Dermatology, The Rockefeller University, New York, NY, USA; 2 Department of Dermatology, Royal Prince Alfred Hospital, New South Wales, Australia

## Abstract

**Background:**

Severe psoriasis vulgaris can be extremely difficult to treat in some patients, even with the newer biological therapies available today.

**Case presentations:**

We present two patients with severe chronic plaque psoriasis who received numerous systemic anti-psoriatic therapies with varied results. Both responded well to initial treatment with efalizumab (anti-CD11a), but then experienced a flare of their disease after missing a dose. However, after disease stablization, both patients responded well to re-introduction of efalizumab, one patient requiring concurrent treatment with infliximab (anti-TNF-α).

**Conclusion:**

These cases are presented to characterize this "flare" reaction, and to inform health care providers that efalizumab can still be administered after disease flare, and again may be a successful therapy.

## Background

Psoriasis may be a long-lasting disease resulting in great morbidity in affected patients. Newer biological therapies may offer a real alternative to those with severe disease, and they are associated with a different toxicity profile than traditional systemic therapies [1]. The agents currently approved by the US FDA are alefacept (anti-CD2, Amevive, Biogen), efalizumab (anti-CD11a, Raptiva, Genentec Inc) and etanercept (anti-TNF receptor, Enbrel, Amgen). Infliximab (anti-TNF-α, Remicade, Centocor) has not yet been approved for psoriasis vulgaris, although it has recently been approved for psoriatic arthritis.

Efalizumab is a humanized monoclonal antibody targeting the α chain of the T cell adhesion integrin lymphocyte function-associated antigen (LFA)-1. The LFA-1- intracellular adhesion molecule (ICAM)-1 interaction plays a crucial role in T cell adhesion at several key points in immune activation pathways. By binding to ICAM-1 on dendritic cell (DCs), endothelial cells and keratinocytes, T cells may be activated, migrate, and interact with keratinocytes respectively. The mechanism of action is not yet completely understood, however during therapy peripheral lymphocytosis is observed, which is most likely due to inhibition of T cell trafficking and blockade of memory T cells entering inflamed skin [2]. Efalizumab is associated with a rebound flare reaction in approximately 5% of patients when therapy is ceased [3]. However, we were not able to find reports of exacerbations of psoriasis while on therapy, as in these cases.

Infliximab is a chimeric anti-TNF-α monoclonal antibody which gives excellent results in the majority of patients at a dose of 5 mg/kg per infusion [4]. Case reports of combination therapies with two biological agents have not yet been reported for psoriasis. The main concern with this therapeutic strategy is the risk of opportunistic infection and malignancy, which should be constantly considered. We present these cases to document the clinical and histological appearance of the flare reaction occurring during previously-effective efalizumab therapy, and demonstrate that this agent can be reintroduced with good clinical effect.

## Case presentations

**Patient 1 **is a 51 year old man from Ecuador, with severe large plaque psoriasis for 15 years, and a strong family history of psoriasis. His medical background included recent-onset hypertension and diabetes, and renal calculi. He takes lisinopril and glyburide, as well as doxepin and atarax when required. His past psoriasis treatments include topical steroids, methotrexate (not tolerated due to nausea), and UVB with minimal effect.

He was first seen at The Rockefeller University, NY, USA, in December 2000 and received numerous courses of biological therapies in the context of our clinical trials program. He initially received efalizumab (100 mg [1 mg/kg] sc weekly for 12 weeks) with good effect. His re-treatment with efalizumab was required in May 2001 because of a sunburn-induced flare, and was permitted under our clinical trial protocol. Another psoaisis flare in Sept 2001 was treated with alefacept (7.5 mg IV for 12 weekly doses) with good effect. Subsequent disease exacerbations were managed well with a course of daclizumab (anti-CD25) therapy, NB-UVB, and cyclosporine.

**Figure 1:**
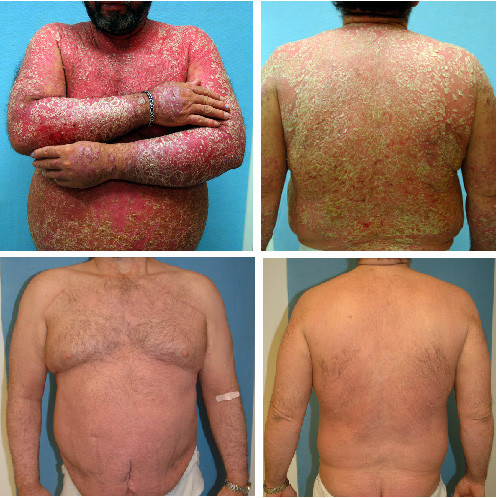
Clinical photos of patient 1 during the flare reaction in June 2004 (upper panels), with follow-up photos after standard-dose treatment with efalizumab for three months (lower panels).

**Figure 2:**
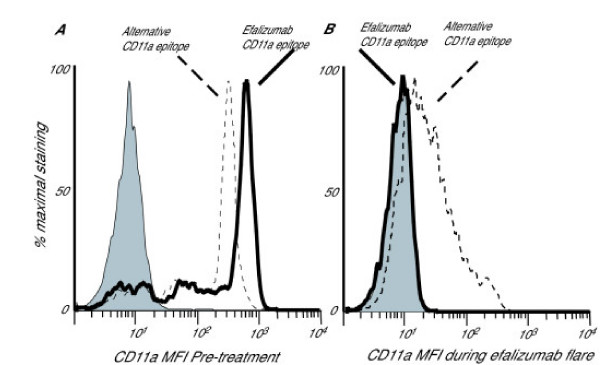
FACS analysis of peripheral blood lymphocytes (gated) of patient 1, pre-treatment (A) and during flare (B). Isotype (Becton Dickenson) is shaded, solid line shows T cell binding of efalizumab-FITC (custom design, Genetech), and dotted line shows binding of an anti-CD11a antibody which binds to a different epitope (clone 25.3, Immunotech). The pre-treatment sample (A) shows high-level binding of both anti-CD11a antibodies. The sample acquired during flare (B) shows the ex-vivo level of binding of efalizumab-FITC is the same as isotype, as there is already saturation of this CD11a epitope by therapeutic administration of efalizumab. There is also reduced binding of the 25.3 epitope indicating significant down-regulation of CD11a surface expression.

Due to previous success with efalizumab and recent USA FDA approval, a disease flare in March 2004 was managed with efalizumab at the standard dose (1 mg/kg/wk sc) at a private clinic. However, he missed a dose in June 2004 and his skin flared again, so he re-attended our clinic (Fig 1). Despite missing a dose, there was still leukocyte CD11a saturation by efalizumab (Fig. 2B, solid line identical to isotype, shaded). At this time, his psoriasis was complicated by Staphlococcal skin infection. To gain control of his skin disease and while waiting for his skin infection to respond to antibiotic treatment (dicloxicillin), he was given low-dose NB-UVB. Efalizumab was re-commenced in September 2004 with good result (110 mg/wk, 1 mg/kg). This has been continued and the patient is currently in remission.

**Figure 3:**
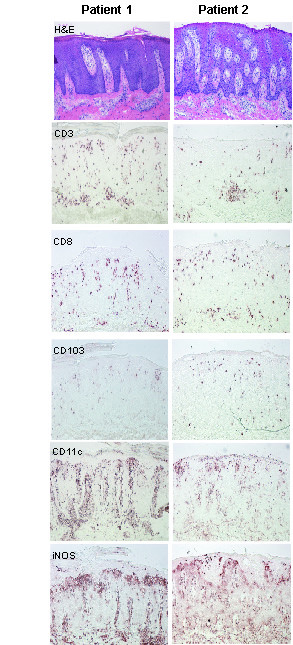
Immunohistochemistry of lesional skin biopsies during psoriatic flare of patient 1 and 2, showing similar features (magnification × 10). Haematoxylin and eosin (H&E) stain showing epidermal hyperplasia, elongation of rete ridges, dilatation of dermal papilla blood vessels, and mononuclear and neutrophil leukocyte infiltration. Some CD3 (BD) T cells infiltrate the dermis and epidermis, and these are mostly CD8^+ ^(BD Pharmingen), although there are less T cells than during untreated psoriasis. Most of the epidermal T cells are CD103^+ ^(Biodesign, Kennebunnk, ME). CD11c^+ ^(BD Pharmingen) and iNOS^+ ^(R&D Systems) cells are dramatically increased compared to non-lesional skin, and stain in a dendritic pattern.

Immunohistochemistry during the flare reaction (Fig. 3), shows there are relatively less lesional CD3^+ ^cells compared to untreated psoriasis, and they are predominantly all CD8^+^. CD103 is also expressed on epidermal T cells. In addition, there are abundant CD11c^+ ^and inducible nitric oxide synthase (iNOS)^+ ^inflammatory cells infiltrating the dermis and epidermis. CD14^+ ^cells are relatively rare (not shown).

**Patient 2 **is a 33 year-old Australian-born Caucasian female with a 14 year history of severe plaque psoriasis, and psoriatic arthritis for 12 years. Her first presentation of psoriasis was with erythroderma at 19 years old. Her past medical history included appendicectomy, infectious mononucleosis, and Chloroquine-resistant *P. falciparum *malaria at age 15 years following travel to Papua New Guinea. She was not taking any medications at the time of initial referral to Royal Prince Alfred Hospital Department of Dermatology, New South Wales, Australia, in 1994.

Past rotational treatments for her psoriasis and psoriatic arthritis included cyclosporine (1990 and 1995), methotrexate (1993 and 1996), acitretin (1994 and 1997), as well as periodic courses of NB-UVB. She was hospitalized in 1999 for erythrodermic psoriasis. In 2001 her psoriasis flared and other therapies tried without success were mycophenolate mofetil, hydroxyurea, tacrolimus, and thioguanine. From July 2001 to November 2002 she received periodic treatment with cyclosporine and acitretin therapy, with poor disease control.

Over the next 2.5 years, this patient was treated with a number of newer biological therapies with standard dosing, sometimes requiring cyclosporine cover and subsequent withdrawal as the patient responded. Etanercept (anti-TNF receptor) (Dec 2002–Feb 2003, 25 mg sc twice week) was initially successful, but was ceased on relapse to erythroderma. Alefacept (anti-CD2) (15 mg IM weekly for 3 months) had no beneficial effect.

Efalizumab (anti-CD11a) (80 mg [1 mg/kg] sc weekly, Dec 2003) induced a dramatic clinical response, and after 6 weeks, cyclosporine was ceased. The patient missed the 18th dose of efalizumab, but the regular dose was given at the subsequent visit. Two days after the "catchup" dose, the patient developed a psoriatic flare involving extensive plaques on all body surfaces. Immunohistochemistry at this time (Fig. 3) shows similar results to patient 1: there are relatively reduced CD3^+ ^and CD8^+ ^cells, epidermal T cells are CD103^+^, and there are abundant CD11c^+ ^and iNOS^+ ^inflammatory cells infiltrating the dermis and epidermis.

During this flare, NB-UVB was administered concomitantly with efalizumab, however a UVB-induced burn was associated with recurrence of her psoriasis. However, by June 2004 there was almost confluent severe plaque psoriasis again with features of erythroderma. The efalizumab dose was increased to 125 mg (1.5 mg/kg) sc weekly, but was ceased in Sept 2004 as there was no further improvement.

Infliximab (anti-TNF-α) (400 mg [5 mg/kg] at 0 and 2 weeks, followed by 400 mg every 6 weeks) was commenced with excellent effect for 4 months, when the patient then experienced another flare of her psoriasis. It was decided to carefully combine infliximab and efalizumab therapy. In February 2005, 62.5 mg (0.7 mg/kg) efalizumab was given sc, and a second dose of 125 mg (1.4 mg/kg) a week later, and she started to respond clinically. She is currently well controlled on this therapy.

## Discussion

We present two cases of severe large plaque psoriasis, with both patients clearing with initial efalizumab treatment, experiencing a flare of their disease after missing a dose of therapy, and responding well to reintroduction of efalizumab after disease stablization. These case reports illustrate that this therapy can be safely reintroduced with good clinical effect in those with limited therapeutic options (Fig. 1), and support our view that this flare reaction is not an allergic hypersensitivity event.

During the flare reaction, flow cytometric analysis of circulating lymphocytes demonstrated that there was persisting saturation of the efalizumab-CD11a epitope, and there was also down-regulation of CD11a expression (Fig. 2). The possible mechanisms of psoriasis flare while on a saturating dose of efalizumab include (1) allergic hypersensitivity, (2) the development of anti-human neutralizing antibodies, (3) lowering of tissue concentration of the drug, or (4) an external trigger inducing other types of leukocytes to enter the skin via an LFA-1-independent mechanism causing inflammation. Safe and effective reintroduction of efalizumab, and the lack of eosinophils on biopsy argue for a non-hypersensitivity mechanism of the flare. If neutralizing antibodies had developed, there would not be efalizumab-CD11a epitope saturation, and further administration would not lead to skin clearing. It is difficult to correlate circulating levels and tissue levels of efalizumab, but it is possible that missing a dose might decrease tissue levels. Finally, we cannot rule out the possibility that entry of non-lymphocyte leukocytes into the skin due to an external trigger such as skin infection might lead to skin inflammation, especially in patient 1. However, the frequency of flare reactions is much less than external events, suggesting that it is an uncommon consequence.

We have also characterized this flare reaction histologically (Fig. 3), demonstrating two main points. In these cases, we found increased CD8^+ ^dominant T cell infiltrates. However, overall there were fewer T cells compared to other psoriasis patients before efalizumab treatment. The CD8^+ ^T cells are located mostly in the epidermis and they express the integrin α_E _(CD103). The α_E _subunit combines with the β7 subunit and binds to E-cadherin on keratinocytes, which mediates epidermal T cell trafficking. Thus CD8^+ ^T cell trafficking during LFA-1 (a β2 integrin) blockade by efalizumab may be regulated by α_E_β7 integrin, permitting epidermal entry of T cells during disease flare.

Secondly, there are abundant CD11c^+ ^and iNOS^+ ^cells in the dermis and epidermis during flare reactions. These markers represent a dendritic cell subset, and may be important in the pathogenesis of psoriasis. These cells may be able to enter the skin via an LFA-1 independent mechanism, and may be playing a direct role in the induction of or maintenance of psoriasis, causing the phenotypic features of erythema and hyperplasia.

Although we would always prefer the use of a single therapeutic agent where possible, the concurrent use of two biologicals of different classes can be considered in those patients with difficult-to-treat severe psoriasis vulgaris with limited therapeutic options. The main reason for caution with combination therapy is that the safety of such combinations is not yet established and so there is limited safety data. One of the main potential risks of such a combination is a decreased response to infection. Patients should be educated about this risk and seek medical attention early if they develop any new symptoms of infection. The main long-term risk of any of the biologicals includes malignancy, and this may be increased when more than one agent is used concurrently. Again, careful regular examination and screening where appropriate is warranted. The length of time a person is on two agents should be tailored to the patient.

## Conclusion

In conclusion, we present two patients who experienced a flare of their severe psoriasis while on initially effective efalizumab therapy. Patient 1 still had saturation of available efalizumab-CD11a sites on circulating lymphocytes, even though he had missed a dose, and was presumably in a therapeutic range. Subsequently, both patients were able to restart efalizumab with effect, one requiring concurrent administration with infliximab. This is reassuring, given the limited therapeutic options for certain psoriasis patients. We have also characterized the histological appearance of this flare reaction, with abundant CD11c^+ ^and iNOS^+ ^DCs, and less CD3, CD8^+ ^and CD103^+ ^cells than are normally seen in psoriasis. These DCs may be playing a critical role in the psoriasis flare and possibly psoriasis.

## List of abbreviations

lymphocyte function-associated antigen-1 (LFA-1)

inducible nitric oxide synthase (iNOS)

intracellular adhesion molecule-1 (ICAM-1)

dendritic cell (DCs)

## Competing interests

JGK has worked as a consultant for Genentech Inc. and Serono. The other authors do not have any financial interest related to this work.

## Authors' contributions

ML drafted the case report of patient 1, analyzed the flow cytometry and immunohistochemistry, and drafted the manuscript. JK supervised treatment of patient 1, analyzed the flow cytometry and immunohistochemistry, and contributed significantly to the manuscript. RB supervised treatment of patient 2, and initiated the effort to characterize the flare reaction and publish the case reports. JT treated patient 2 and drafted the case report of patient 2. All authors read and approved the final manuscript.

## Pre-publication history

The pre-publication history for this paper can be accessed here:


